# Quantum measurement optimization by decomposition of measurements into extremals

**DOI:** 10.1038/s41598-020-65934-w

**Published:** 2020-06-10

**Authors:** Esteban Martínez-Vargas, Carlos Pineda, Pablo Barberis-Blostein

**Affiliations:** 1 Física Teòrica: Informació i Fenòmens Quàntics, Departament de Física, Universitat Autònoma de Barcelona, 08193 Bellaterra, Barcelona Spain; 2 Instituto de Física, Universidad Nacional Autónoma de México, México City, CDMX 01000 Mexico; 3 Instituto de Investigaciones en Matemáticas Aplicadas y en Sistemas, Universidad Nacional Autónoma de México, México city, CDMX 01000 Mexico

**Keywords:** Quantum metrology, Quantum information

## Abstract

Using the convex structure of positive operator value measurements and several quantities used in quantum metrology, such as quantum Fisher information or the quantum Van Trees information, we present an efficient numerical method to find the best strategy allowed by quantum mechanics to estimate a parameter. This method explores extremal measurements thus providing a significant advantage over previously used methods. We exemplify the method for different cost functions in a qubit and in a harmonic oscillator and find a strong numerical advantage when the desired target error is sufficiently small.

## Introduction

One of the goals of metrology is to provide an optimal strategy to measure the value of a parameter under certain fixed conditions. For completeness, both an approximate value of the parameter and an estimation of the error must be given. If the physical system from which the parameter is to be estimated is analyzed within the framework of quantum mechanics, we shall speak about *quantum metrology*^1–5^. Motivated by the fact that quantum systems can offer an important advantage over classical systems in precision when estimating a parameter^6^, there has been intense theoretical and experimental advances in the area in the last years^7,8^. Moreover, precision in the estimation of parameters has several applications in the development of quantum technologies^9,10^ and quantum state manipulation^11^.

To estimate a parameter of a physical system one acquires data through measurements; the estimation of the parameter is obtained by applying a function, known as the estimator, to the data. The probability distribution of measurement outcomes can be modelled using a statistical model of the experiment: the probability distribution of outcomes conditioned to the value of the parameter. This statistical model might describe, say, a noisy measurement apparatus. In this article we specialize to the case of quantum mechanics, where the probability distribution is given by the Born rule and the statistical model is obtained once it is decided which operator is going to be measured. In addition to the random component of measurement, we also consider classical noise, which we include through the density matrix formalism.

The cost function that quantifies the error of the parameter estimation of a quantum system (for example the mean square error) depends on both the measurement to be performed and the estimator; the optimal measurement strategy consists of the quantum measurement and the estimator that extremizes it. However, finding the extreme of a cost function over all possible quantum measurements and estimators is not simple. Alternatively, when a Cramér-Rao type inequality exists, the problem can be reduced to find the extreme of another cost function (for example the Fisher information) over all the quantum measurements. This simplifies the problem because it is not longer necessary to maximize over the space of estimators. One still has to deal with extremizing a cost function over all quantum measurements, which in general is difficult. However, under special circumstances, such as symmetries, the problem can be simplified^12^.

It is possible, though very costly, to numerically find the maximum over all quantum measurements of cost functions. The straightforward way to solve the problem is to randomly sample the space of all *positive operator value measures* (POVMs), evaluate the cost function on this sample, and keep the maximum value obtained. This method, that we call the *random sampling method* (RSM), is very inefficient since the POVM space is large.

In this paper, we show how to numerically find the maximum over all quantum measurements of the Fisher information and the Bayesian version of this bound, the Van Trees bound^13^, in a way that is orders of magnitude faster than using the RSM. We rely on the following: (i) the cost functions of interest in quantum metrology are convex with respect to the POVMs and (ii) the set of POVMs is convex^14^. Therefore, following the maximum principle^15,16^, the maximum over the POVMs must be on an extremal point of the POVM set. Our approach is simply to sample randomly extremal POVMs and get the highest value of the appropriate cost function. It is easy to produce efficiently a general POVM from a random unitary matrix, however, it is not trivial to produce randomly extremal POVMs. For this we used the algorithm proposed by Sentís *et al*.^17^.

We call our method the *random extreme sampling method* (RESM). The techniques presented here can be used, for example, to find numerically the maximum value of the quantum Van Trees information^18^, the quantum Fisher information (even if the input state is not pure), or any convex cost function. Together with the maximum, the corresponding quantum measurement is obtained. The method can also be applied to the case of multivariable convex cost functions, and thus used to find the optimal measurement in the estimation of several parameters. For example: Given a multivariable statistical model we can construct the Fisher matrix, its diagonal elements gives a bound on the variance of each parameter. We can use our method to find the quantum measurement that maximizes any convex cost function of the diagonal elements.

Our approach can also be used in other areas different from quantum metrology, where a maximization over POVMs is required. An example is statistical decision theory^19^. The problem is the following: we have a set of possible decisions, from which one is chosen depending on the outcome of a quantum system measurement. The distribution probability for the decisions is given by a quantum measurement. We look for the best decision. How good is the decision is rated by a cost function defined in the space of quantum measurements. The problem of finding the best decision is mapped to the problem of maximizing this cost function over all the quantum measurements. Note that quantum parameter estimation can be cast as a problem of statistical decision theory, where the decision to be chosen is an estimation of the parameter.

## Results

In this section we first introduce some convex cost functions that give bounds to the error of parameter estimation. Then we present our main result: a numerical algorithm that allow us to find the maximum over all quantum measurement of any convex cost. We finish with examples of interest where we apply the algorithm.

### Bounds in the error of parameter estimation

We discuss how to get bounds for the error made in an estimation process. We use two error measures: the mean squared error and the Bayesian mean squared error, which is used when some information is known about the parameter. This discussion is general and just assumes that a statistical model is given. We also discuss how to apply these ideas for a quantum system.

#### Crámer-Rao inequality

In this section, we introduce some basic quantities needed to develop further the discussion. Let

1 $$p({\bf{y}}|\theta )$$

be the distribution probability of the outcomes ${\bf{y}}$, of the random variable ${\bf{Y}}$, conditioned to a fixed value of the real parameter $\theta$. We assume that each ${\bf{y}}$ is a set of real numbers of fixed finite size. The function $p({\bf{y}}|\theta )$ is the statistical model; ${\bf{y}}$ represents what is measured in an experiment and its probability distribution, $p$, depends on the parameter $\theta$. Using the outcomes ${\bf{y}}$, the parameter is estimated through the real-valued function $\hat{\theta }({\bf{y}})$, known as the estimator. The estimator is unbiased if it is on average correct, meaning that its expected value is equal to the actual value of the parameter,

2 $$\langle \hat{\theta }({\bf{y}})\rangle =\int \,{\rm{d}}{\bf{y}}\,p({\bf{y}}|\theta )\hat{\theta }({\bf{y}})=\theta \mathrm{}.$$

The uncertainty of the estimator is given by the mean squared error, defined as

3 $${\varsigma }^{2}\equiv \int \,{(\hat{\theta }({\bf{y}})-\theta )}^{2}p({\bf{y}}|\theta ){\rm{d}}{\bf{y}}\mathrm{}.$$

We say that the measurement strategy is optimal if the estimator minimizes the mean squared error. Finally, let us define the Fisher information

4 $$F(\theta )\equiv \int \,{\left(\frac{\partial \mathrm{ln}p({\bf{y}}|\theta )}{\partial \theta }\right)}^{2}p({\bf{y}}|\theta ){\rm{d}}{\bf{y}}.$$

If the estimator is unbiased (i.e. if Eq. (2) holds), using the Cauchy-Schwarz inequality, one arrives to the Cramér-Rao inequality^20,21^:

5 $${\varsigma }^{2}F(\theta )\ge 1.$$

Note that ${\varsigma }^{2}$ depends on the choice of the specific estimator $\hat{\theta }({\bf{y}})$, whereas the Fisher information depends only on the distribution probability of the random variable. From the Cramér-Rao inequality, we see that the inverse of the Fisher information bounds from below the mean squared error independently of the estimator we use: the larger the Fisher information the smaller the error bound. Fisher showed that in the limit where the number of measurements goes to infinity, the maximum likelihood estimator saturates this inequality^22^.

#### Quantum Cramér-Rao inequality

We want to find the best measurement strategy to estimate a parameter, $\theta$, that appears in the Hamiltonian of a quantum system. In order to estimate the parameter we proceed as follows: we start with an initial state and let the system evolve some *fixed* time. The dynamics of the system depends on the parameters of the Hamiltonian; after the evolution, the state of the system depends on the parameter we want to estimate: $\rho =\rho (\theta )$. We then measure some observable of the system and use the result to estimate $\theta$. Fixing the Hamiltonian, time and initial state, we want to know if the strategy we are using minimizes the error in the parameter estimation.

General measurements in quantum mechanics are described by the positive operator valued measure (POVM) formalism, which we briefly recall in order to fix the notation. If $\{E(\xi )\}$ is a POVM parametrized by the real parameter $\xi$, for each value of $\xi$, $E(\xi )$ is a self-adjoint operator on the system Hilbert space. They satisfy the completeness relation

6 $$\int \,\hat{E}(\xi ){\rm{d}}\xi =1,$$

and the probability of measuring the result $\xi$ is

7 $$p(\xi |\theta )=Tr(\rho (\theta )E(\xi \mathrm{))}.$$

In order for Eq. (7) to be a probability distribution we require that the elements $E(\xi )$ to be positive semidefinite,

8 $$E(\xi )\ge 0.$$

Notice that $\xi$ can also belong to a finite set (or a combination of several discrete and continuous indices) if the number of possible outcomes is finite. The expressions throughout this article generalize replacing $\int \,{\rm{d}}\xi$ by ${\sum }_{\xi }$.

Fixing the POVM and thinking of (7) as the distribution probability of the outcomes [as in Eq. (1)], one can use the tools introduced in Section (2.1.1). In particular, we can calculate the Fisher information and use the Cramér-Rao inequality to know if a given estimator is optimal. However, note that there is a dependence of the Fisher information on the POVM we choose. In order to have the lowest bound for the error, we maximize the Fisher information over all the possible measurements^23^

9 $${F}_{Q}(\theta )=\mathop{{\rm{\max }}}\limits_{\{\hat{E}(\xi )\}}\,\int \,{\left(\frac{\partial \mathrm{ln}p(\xi |\theta )}{\partial \theta }\right)}^{2}p(\xi |\theta ){\rm{d}}\xi \mathrm{}.$$

The quantity ${F}_{Q}(\theta )$ is known as the quantum Fisher information, and through the Cramér-Rao inequality,

10 $${\varsigma }^{2}{F}_{Q}(\theta )\ge 1,$$

tells us the minimal possible error for the best measurement strategy for estimating a parameter appearing in the Hamiltonian of a quantum system. Equation (10) is a direct result from Eq. (5). Since Eq. (5) is valid for every POVM, it is valid for the one in which the maximum Fisher information is attained. The POVM that maximizes ${F}_{Q}$ is the one that should be used to get the smallest error in the parameter estimation^1^; we call this POVM the optimal POVM. If the quantum state is pure there are analytical formulas for finding ${F}_{Q}$. Otherwise no general formula is known and one must rely in numerical methods.

#### Bayesian Cramér-Rao inequality

We consider the case where we have some partial knowledge of the parameter to be estimated. An example: We want to estimate the velocity of one particle in a dilute gas at temperature $T$. Without doing any measurement, we know that the particle velocity can be interpreted as a random variable that satisfies the Maxwell-Boltzmann distribution. Note that with the information we already have, we can estimate the velocity as the mean of the Maxwell-Boltzmann distribution $v=\sqrt{8{K}_{b}T/(m\pi )}$ with a variance $\mu ={K}_{b}T(3\pi -8)/(m\pi )$. Here ${K}_{b}$ is the Boltzmann constant and $m$ the mass of the particle. It is reasonable to design the experiment to measure velocities around $v$. The result of the measurement should improve the estimation, giving an error smaller than $\mu$. Note that, in general, experiments designed to measure a parameter usually work for some expected range of its value, which implies assumptions were taken about the value of the parameter.

The Bayesian Cramér-Rao inequality can be used to decide what is the best estimator in the situation where we have partial knowledge of the parameter. We model the parameter as the random variable $\Theta$, with outcomes $\theta$, and probability distribution $\lambda (\theta )$. The outcomes of the experiment are modelled as the random variable ${\bf{Y}}$, with outcomes ${\bf{y}}$, and probability distribution $p({\bf{y}}|\theta )$. The cost function we want to minimize is the mean square error

11 $${\Xi }^{2}=\int \,{(\hat{\theta }({\bf{y}})-\theta )}^{2}P({\bf{y}},\theta ){\rm{d}}\theta {\rm{d}}{\bf{y}},$$

where $$P({\bf{y}},\theta )=p({\bf{y}}|\theta )\lambda (\theta )$$.

It can be shown that the error is bound from below by the Cramér-Rao type inequality^13^,

12 $${\Xi }^{2}Z\ge \mathrm{1,}$$

where the generalized Fisher information, $Z$, can be written as

13 $$Z=\int \,F(\theta )\lambda (\theta ){\rm{d}}\theta +\int \,{\left(\frac{\partial \mathrm{ln}\lambda (\theta )}{\partial \theta }\right)}^{2}\lambda (\theta ){\rm{d}}\theta \mathrm{}.$$

The first term of the sum is the expectation value of the Fisher information; the second term is the Fisher information of the probability distribution of the possible values of the parameter. The last term codifies what we already know about the parameter. As can be seen from the previous equation, the generalized Fisher information is larger than the Fisher information due to the knowledge we already have of the parameter. This has a simple interpretation: we can use $\lambda (\theta )$ to estimate the parameter, and measuring the system necessarily diminishes the error in the estimation of the parameter. The best strategy for measuring a parameter, with known information codified in a probability distribution, is given by the estimator, $\hat{\theta }$, that saturates inequality (12).

If we want to estimate the outcomes of a random variable, the problem of finding the best strategy is exactly the same as discussed in this section. In this case the experiment is repeated several times with the values of the parameter satisfying the probability distribution of the random variable. An example: measure the velocity of several particles in thermal equilibrium in a dilute gas. The velocities for the classical particles obey a Maxwell-Boltzmann distribution.

In this context, we found useful^24^, a review of bayesian inference in physics.

#### Bayesian quantum Crámer-Rao inequality

We want to estimate the parameter $\theta$ of a Hamiltonian in a quantum system where the known information about $\theta$ is codified in the probability distribution $\lambda (\theta )$. Given a POVM, $\{E(\xi )\}$, a statistical model can be built through Eq. (7), and the problem is reduced to the classical one.

The POVM, $\{{E}_{{\rm{\max }}}(\xi )\}$, that maximizes the generalized Fisher information (13), together with the appropriate estimator, saturates the Cramér-Rao type inequality

14 $${\Xi }^{2}{\tilde{Z}}_{Q}\ge 1,$$

where

15 $${\tilde{Z}}_{Q}=\mathop{{\rm{\max }}}\limits_{\{\hat{E}(\xi )\}}\left(\int \,{\left(\frac{\partial \mathrm{ln}p(\xi |\theta )}{\partial \theta }\right)}^{2}P(\xi ,\theta ){\rm{d}}\theta {\rm{d}}\xi \right)+\int \,{\left(\frac{\partial \mathrm{ln}\lambda (\theta )}{\partial \theta }\right)}^{2}\lambda (\theta ){\rm{d}}\theta \mathrm{}.$$

We call ${\tilde{Z}}_{Q}$ the quantum Van Trees information.

If we want to minimize the error in the parameter estimation, and we codify what we know about the parameter in the probability distribution $\lambda (\theta )$, we have to implement the quantum measurement given by $\{{E}_{{\rm{\max }}}(\xi )\}$^18^.

### Numerical algorithm

The calculation of cost functions such as ${F}_{Q}$ or ${\tilde{Z}}_{Q}$ is not easy, as it implies an optimization over all POVMs. In this section, we present our main result: an efficient numerical procedure to calculate the maxima, over all POVMS, of convex cost functions.

#### Convexity

The quantum Van Trees information is convex; this follows directly noticing that the set of POVMs^14,25^ and the Fisher information are convex. Fisher information can be rewritten as $F=\int \,{(p{\prime} )}^{2}/pd{\bf{x}}$, where the prime indicates the derivative with respect to $\theta$. It then follows that

16 $$\begin{array}{c}\frac{1}{2}\,\int \,\frac{{({p{\prime} }_{1})}^{2}}{{p}_{1}}{\rm{d}}{\bf{x}}+\frac{1}{2}\,\int \,\frac{{({p{\prime} }_{2})}^{2}}{{p}_{2}}{\rm{d}}{\bf{x}}-\int \,\frac{{[({p{\prime} }_{1}+{p{\prime} }_{2}\mathrm{)/2]}}^{2}}{({p}_{1}+{p}_{2}\mathrm{)/2}}{\rm{d}}{\bf{x}}\\ \,=\,\frac{1}{2}\,\int \,\frac{1}{{p}_{1}{p}_{2}({p}_{1}+{p}_{2})}{[{p{\prime} }_{1}{p}_{2}-{p}_{1}{p{\prime} }_{2}]}^{2}{\rm{d}}{\bf{x}}\ge 0,\end{array}$$

provided that ${p}_{1,2}\ge 0$. For a combination with other weights, continuity and a recursive procedure imply convexity of *F*. From these two observations, we infer that the Van Trees information is also convex, as the integral of convex functions is also convex.

Since the maximum of the convex cost functions lies on the extremal points of all POVMs, we only need to search in this subset simplifying greatly the optimization task. A way to sample randomly such a set is presented in the following sub-section.

#### The algorithm

The outline of the algorithm is as follows: We produce a random POVM, and decompose it in extremals. We then evaluate the cost function using extremal POVMs and choose the one which yields the highest value. We repeat the procedure several times and keep the optimal POVM. We provide an implementation in an online repository^26^.

##### Random Sampling Method

To produce a random POVM, we use the purification algorithm^27^ backwards, which transforms a general POVM into a usual projective measurement in an enlarged space.

The first step is to produce a random unitary matrix that acts on both the original space and an ancilla Hilbert space. The dimension of this ancilla space is the number of outputs of the initial POVM. Because the extremal POVMs have at most ${d}^{2}$ elements, where $d$ is the Hilbert space dimension^14^, nothing is gained if the dimension of the ancilla space is larger than ${d}^{2}$. For example, if we are trying to estimate a parameter of a qubit, we only need to use a dimension 2 Hilbert space and a dimension 4 ancilla space. If the Hilbert space is large or has an infinite dimension, we run the algorithm for some arbitrary dimension of the ancilla space and then increase its dimension until stable results are obtained.

The aforementioned unitary matrix is chosen with a measure invariant with respect to multiplication by unitary operators, i.e., with the Haar measure. The ensemble induced by the aforementioned measure is called the *circular unitary ensemble* (CUE)^28^. The easiest way to construct a representative member of the CUE is to construct a member of the *Gaussian unitary ensemble* (GUE)^28^, the ensemble of hermitian matrices invariant under unitary conjugation subject to the condition that the ensemble average of the trace of the square of the matrices is fixed. Generating a member $H$ of such an ensemble is simple: we build a matrix $A$ with Gaussian complex numbers, all with equal standard deviation and zero mean. Let $H=A+{A}^{\dagger }$, we can then calculate the matrix elements of $UH{U}^{\dagger }$, for any unitary $U$, and verify that the distribution of all matrix elements of the rotated matrix remains invariant. Thus, the eigenbasis of $H$ has the Haar measure, and then the matrix $U$ that diagonalizes $H$ is a member of the CUE with the appropriate measure. The routine CUEMember calculates such an operator and can be found in^26^.

It is well known that we can interpret an arbitrary $n$ output POVM as a projective measurement in a space built from the original one and a coupled $n$-dimensional ancilla space. If $\{|\mu \rangle {\}}_{\mu =1,\ldots ,n}$ is an orthonormal base in the ancilla space, $|\Psi \rangle$ a state in the original Hilbert space, and $\{{Q}_{m}\}$ the operators characterizing the POVM, we can define a unitary operator $U$ such that

17 $$U|\Psi \rangle |1\rangle =\sum _{m}\,{Q}_{m}|\Psi \rangle |m\rangle ,$$

based on the completeness relation ${\sum }_{m}\,{Q}_{m}^{\dagger }{Q}_{m}=1$. Moreover, the projective measurement defined by the projectors $\{1\otimes |m\rangle \langle m\rangle {\}}_{m=1,\ldots ,n}$ is equivalent to the measurement defined by $\{{Q}_{m}\}$, in the sense that the probabilities are the same, and the states, after discarding the ancilla space, are also identical, see^27^. Equation (17) can also be interpreted as a way to induce a POVM measurement in the original Hilbert space, starting from a unitary operator in an extended Hilbert space. In fact, if we replace $|\Psi \rangle$ by the basis state $|j\rangle$ and premultiply by the bra $\langle i|\langle m|$, we obtain

18 $$\langle i|{Q}_{m}|j\rangle =\langle i|\langle m|U|j\rangle |1\rangle .$$

Thus, from the random unitary $U$ we can get all matrix elements of each of the ${Q}_{m}$, according to Eq. (18). Since for all POVMs one can build a unitary transformation in the extended space such that Eq. (18) holds^27^, sampling all unitaries in the extended space guaranties sampling all POVMs with the corresponding number of outcomes. The routine POVM calculates the POVM in this way and can be found in^26^. The aforementioned method to sample POVMs will be called *Random sampling method*, or RSM for short.

###### Naimark dilation

At this point, we would like to mention another POVM sampling method, inspired in Naimark’s dilation theorem^29^.

We start with a unitary matrix, acting on the original space tensored with an ancilla space ${ {\mathcal H} }_{{\rm{ancilla}}}$. The columns $|{v}_{i}\rangle$ of this matrix can be interpreted as an orthonormal basis on the extended space of dimension *d*′. Notice that *d*′ is a multiple of *d*, the dimension of the original Hilbert space. Let us define the *d*′ operators

$${M}_{i}={{\rm{Tr}}}_{{ {\mathcal H} }_{{\rm{ancilla}}}}|{v}_{i}\rangle \langle {v}_{i}|\frac{{\bf{1}}}{d}\otimes |r\rangle \langle r|,$$

where $|r\rangle$ is a random state on the ancilla space. Notice that $\langle \Psi |{M}_{i}|\Psi \rangle =|\langle {v}_{i}|(|\Psi \rangle \otimes |r\rangle {|}^{2}$, the probability of projecting to $|{v}_{i}\rangle$ the state $|\Psi \rangle \otimes |r\rangle$, so all ${M}_{i}$ are semipositive defined operators. Moreover, they inherit the completeness relation ${\sum }_{i}\,{M}_{i}={\bf{1}}$ from the completeness relation $|{v}_{i}\rangle \langle {v}_{i}|={\bf{1}}$ of the orthonormal basis in the enlarged space. Thus, $\{{M}_{i}\}$ form a POVM of *d*′ outcomes.

If we build a POVM with the aforementioned recipe, with the unitary matrix chosen from the CUE and the state $|r\rangle$ with the Haar measure, we say that we are sampling a POVM with the ND method.

##### Conversion to a rank-1 POVM

To proceed further, we need a rank-1 POVM, so we must transform the aforementioned POVM accordingly. Recall that a rank-1 POVM is one whose elements are all rank-1 operators. However, typically, the $\{{Q}_{m}\}$ are not rank-1 operators.

For any given $\tilde{Q}\in \{{Q}_{m}\}$ that is not a rank-1 operator, we perform the spectral decomposition ${\tilde{Q}}^{\dagger }\tilde{Q}={\sum }_{i=1}^{l}={\lambda }_{i}{\tilde{Q}}_{i}$ using a standard algorithm. Notice that the ${\tilde{Q}}_{i}$ are projectors, and ${\lambda }_{i} > 0$. We then replace $\tilde{Q}$ with the $l$ operators $\sqrt{{\lambda }_{i}}{\tilde{Q}}_{i}$, and the completeness relation remains valid since ${\sum }_{i}\,{(\sqrt{{\lambda }_{i}}{\tilde{Q}}_{i})}^{\dagger }\sqrt{{\lambda }_{i}}{\tilde{Q}}_{i}={\tilde{Q}}^{\dagger }\tilde{Q}$. Notice that the number of elements at the end of the algorithm will be larger than the number of outputs of the initial POVM.

This check and the corresponding transformation of the POVMs are done with the routines projector and eivalues.

Random extremal sampling method Let us define ${a}_{i}={\rm{Tr}}\,{Q}_{i}$, and ${A}_{ij}={a}_{j}^{-1}{\rm{Tr}}({Q}_{j}{G}_{i})$ with $\{{G}_{j}\}$ an orthonormal traceless base for hermitian matrices of the appropriate dimension. In our case, we used the Gell-Mann matrices. We also define ${A}_{{d}^{2},j}=1$, so that the completeness condition over POVMS reads

$$Aa=b,$$

if we define the *d*^2^-dimensional vector $b=(0,\ldots ,0,d)$. We now propose the linear program

19 $$\begin{array}{llll}{\rm{find}} & x &  & \\ {\rm{subject}}\,{\rm{to}} & Ax & = & b,\,x\ge 0.\end{array}$$

Even though $x=a$ is a solution to the problem, the usual numerical algorithms provide an extremal point. Notice that if an element ${x}_{i}$ of the solution is 0, it means that we do not include the operator in the POVM. The construction of the matrix $A$ and vector $b$ is done with the routine AConstruction. Let this extremal solution be ${x}^{{\rm{ext}}}$.

 The Mathematica LinearProgramming[c,A,b] implementation solves the following linear program:

20 $$\begin{array}{llll}{\rm{minimize}} & {c}^{T}x &  & \\ {\rm{subject}}\,{\rm{to}} & Ax & = & b,\,x\ge 0,\end{array}$$

where *c* is a constant vector. As we are interested only in *finding* a solution and not in optimizing a given vector, we give a random vector *c* to the usual Mathematica LinearProgramming routine. This vector *c* has random entries between 0 and 1 given by the uniform distribution with the Mathematica routine RandomReal. This process is done by our routine LinearProg.

To obtain the extremal POVM we start by defining *x*′ via

21 $$a=p{x}^{{\rm{ext}}}+\mathrm{(1}-p)x{\prime} ,$$

with *p* a scalar. Requiring that $x{\prime} \ge 0$ can be enforced letting

22 $$p=\mathop{{\rm{\min }}}\limits_{i}\frac{{a}_{i}}{{x}_{i}^{{\rm{ext}}}},$$

which in turn implies that *p* is a probability and that for some $i$, ${x{\prime} }_{i}=0$. If we define ${{\bf{Q}}}^{{\rm{ext}}}={x}^{{\rm{ext}}}{\bf{Q}}$ and ${\bf{Q}}{\prime} =x{\prime} {\bf{Q}}$, we can write

$${\bf{Q}}=p{{\bf{Q}}}^{{\rm{ext}}}+(1-p){\bf{Q}}{\prime} .$$

Indeed, ${{\bf{Q}}}^{{\rm{ext}}}$ is an extremal POVM^17^, and since one of the elements of *x*′ is null, ${\bf{Q}}{\prime}$ is a $n-1$ output POVM (given that **Q** is an $n$ output POVM) for which we can iterate the algorithm until a single output POVM is obtained. Notice that with this algorithm, all POVMs with a given number of outputs can in principle be sampled.

The routine BuildExtremal constructs an extremal POVM from the output of the Linear Program. The routine CalculateP calculates the $p$ probability from Eqs. (21) and (22). Finally, the auxiliary solution ${\bf{Q}}{\prime}$ is built with the routine AuxiliarSol.

### Examples

In this section we apply the method described in the section **Numerical algorithm** to estimate the quantum Fisher information and the quantum Van Trees information. In the examples we observe a computational speedup when using the algorithm presented here compared with methods that randomly sample the whole POVMs space.

#### Qubit

We use the algorithm to calculate the quantum Fisher information for estimating a parameter encoded in a pure qubit state. We use known analytical results to benchmark our numerical method.

We consider a spin 1/2 particle in the state,

23 $$|\Psi (\theta ,\eta )\rangle =(\begin{array}{c}{e}^{-i\theta /2}\,\cos (\eta /2)\\ {e}^{i\theta /2}\,\sin (\eta /2)\end{array}),$$

where $\theta \in [0,2\pi )$ is the phase between the two basis states and $\eta \in [0,\pi ]$ is a known parameter that characterizes the weight of each element of the superposition. The problem is the following: we want to find the best strategy to estimate the phase $\theta$ given that $\eta$ is known^30^.

Given a set of states parametrized by $\xi$, i.e. $\rho (\xi )$, we consider the following family of POVMs:

24 $${\bf{P}}(\xi )=\{\rho (\xi ), {\mathcal I} -\rho (\xi \mathrm{)\}}.$$

Each of these POVMs has two elements that corresponds to the outcomes $1$ and $2$. In this subsection we shall consider the particular case

25 $$\rho (\xi )=|\Psi (\xi ,\eta )\rangle \langle \Psi (\xi ,\eta )|.$$

Using Eq. (7) we find that the probability of measuring outcome 1 or 2 for the POVM $\xi$ is given by

26 $$\begin{array}{rcl}{p}_{\xi }(1|\theta ) & = & \langle \Psi (\theta ,\eta )|\Psi (\xi ,\eta )\rangle \langle \Psi (\xi ,\eta )|\Psi (\theta ,\eta )\rangle ,\\ {p}_{\xi }(2|\theta ) & = & 1-{p}_{\xi }(1|\theta ).\end{array}$$

The Fisher information for this probability distribution is

27 $${F}^{(\xi ,\eta )}(\theta )=\frac{{\sin }^{2}(\eta )}{1+{\cos }^{2}(\eta )\,{\tan }^{2}((\xi -\theta )/2)},$$

which is a function of the parameter we want to estimate, i.e. $\theta$. Notice that there is a dependence on the initial state, via $\eta$, and on the POVM used, via $\xi$; we make these dependencies on the POVM explicit via a superscript. When the state is pure, the maximum quantum Fisher information can be analytically calculated^23^. In this example the quantum Fisher information is the maximum of ${F}^{(\xi ,\eta )}(\theta )$ with respect to $\xi$:

28 $${F}_{Q}(\theta )=\mathop{{\rm{\max }}}\limits_{\xi }\,{F}^{(\xi ,\eta )}(\theta )={\sin }^{2}(\eta ).$$

**Figure 1:**
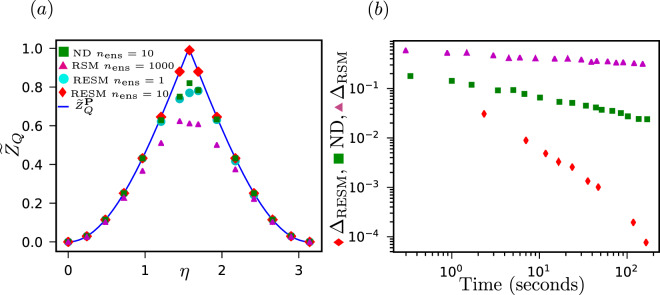
We study the numerical accuracy (**a**) and time cost (**b**) of estimating the quantum Van Trees information, for a qubit encoding parameter $\theta$ on its phase, see Section 2.3.1. (**a**) A numerical estimation was done using the RSM method with ${n}_{{\rm{ens}}}=10$ (green triangles) and ${n}_{{\rm{ens}}}=1000$ (purple triangles) samples and using the REMS method with ${n}_{{\rm{ens}}}=1$ (cyan circles) and ${n}_{{\rm{ens}}}=10$ (red diamonds) samples. The ansatz (29), resulting in Eq. (30), is shown as a blue line. We consider different families of states parametrized by $\eta$, see Eq. (23). The number of outcomes of the POVM is fixed to 4, as this is the maximum number of outcomes for an extremal POVM in a 2-dimensional Hilbert space^25^. Note that the RESM method gives much better results with one sample than the RSM method with 1000. (**b**) Error in the numerical estimation of ${F}_{Q}$, see Eq. (28), sampling directly the whole space of POVMs (RMS) or only its extremal points (RESM) for $\eta =\pi /2$, with respect to the computational time invested. The slope for RSM case is ${m}_{{\rm{RSM}}}=-\,0.63$ and for RESM is ${m}_{{\rm{RESM}}}=-\,1.84$. The error with the method proposed decreases much faster using RESM rather than RMS.

In order to evaluate the performance of the proposed algorithm, we apply the RSM and RESM methods and compare their performance with the exact result (28). We define the errors

$$\begin{array}{rcl}{\Delta }_{{\rm{RSM}}} & = & {F}_{Q}(\theta )-{F}_{{\rm{RSM}}},\\ {\Delta }_{{\rm{RESM}}} & = & {F}_{Q}(\theta )-{F}_{{\rm{RESM}}},\end{array}$$

where *F*_RSM_ and *F*_RESM_ are the Fisher information numerically calculated using the RSM and RESM respectively. In Fig. 1(b), we plot running time vs error for the two methods. It is clear from the plot that RESM is better, and the longer the program runs, the better the results using RESM compared with RSM. For this example, we obtain an error two orders of magnitude smaller running the program for the same length of time.

Now we consider that we have some information about $\theta$ codified in the probability distribution $p(\theta )$; limits to the error in the estimation are given by the Cramér-Rao type inequality (14). We assume that the angle $\theta$ has the uniform distribution $p(\theta )=1/2\pi$ in Eq. (29). First we consider the maximization of the generalized Fisher information over the family of POVMs, ${\bf{P}}(\xi )$, given by Eqs. (24) and (25)

29 $${\tilde{Z}}_{Q}^{{\bf{P}}}=\mathop{{\rm{\max }}}\limits_{\xi }\,\int \,{\rm{d}}\theta {\rm{p}}(\theta ){{\rm{F}}}^{(\xi ,\eta )}(\theta \mathrm{)}.$$

Because we are using a subset of all the POVMS ${\tilde{Z}}_{Q}^{{\bf{P}}}\le {\tilde{Z}}_{Q}$, this approach allows us to get an analytic approximation for the quantum Van Trees information. For a uniform superposition ($\eta =\pi /2$), the Fisher information becomes independent of $\xi$; in fact ${\tilde{Z}}_{Q}^{{\bf{P}}}={\tilde{Z}}_{Q}=1$, see Eq. (27). This implies that any POVM from the family ${\bf{P}}(\xi )$ maximizes the Fisher information. In general we obtain

30 $${\tilde{Z}}_{Q}^{{\bf{P}}}(\eta )=1-|\,\cos (\eta )|,$$

so we can assert that if only POVMs of the family ${\bf{P}}(\xi )$ are allowed, the best estimation is in the case where $\eta =\pi \mathrm{/2}$.

Now we apply RESM to calculate ${\tilde{Z}}_{Q}$ and compare with ${\tilde{Z}}_{Q}^{{\bf{P}}}(\eta )$, see Fig. 1(a). The maximum of ${\tilde{Z}}_{Q}$ is again obtained when $\eta =\pi /2$. That means that the lowest error in the phase estimation is obtained when the weights of the superposition are the same. The figure suggests that ${\tilde{Z}}_{Q}^{{\bf{P}}}={\tilde{Z}}_{Q}$.

We observed that surprisingly, almost any extremal POVM is useful for finding the maximum ${\tilde{Z}}_{Q}$. We require very few samplings (≈10) to observe good agreement between Eq. (30) and the numerical calculations. We also arrive at a reasonable estimate with 1 sample for most $\eta$s.

#### Phase estimation

We calculate the Van Trees information for the phase estimation problem, one of the workhorses of quantum metrology. Since for this case no analytical solutions are known, this is an interesting testbed for our method.

##### Initial coherent state

We want to estimate the phase difference $\theta$ between two paths that light can follow, see^18^ for a similar calculation. We probe the system with a coherent state, such that one path yields the state $|\alpha \rangle$ (with *α* a complex number) and the other

31 $$|\phi (\theta )\rangle ={e}^{i\hat{n}\theta }|\alpha \rangle =|{e}^{i\theta }\alpha \rangle ,$$

where $\hat{n}$ is the number operator.

**Figure 2:**
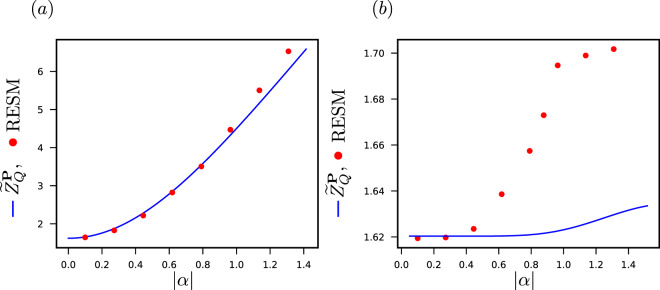
(**a**) Quantum Van Trees information for estimating a Gaussian distributed random phase acquired by a coherent state. (**b**) Calculation of ${\tilde{Z}}_{Q}$ for a displaced thermal state with $\alpha =r\times (1+i)/\sqrt{2}$ and $r\in  {\mathcal R}$ (we varied $r$) with a random phase chosen from Gaussian distribution. It can be seen how the ansatz does not work. For the figure (**a**) we sampled 150 times, we considered a POVM with 10 outcomes and we truncated the photon space to the lowest seven states of the harmonic oscillator. For the figure (**b**) we sampled 200 times and the approximation of the dimension of the Hilbert space and the number of outcomes was the same, the dimensionless temperature is ${k}_{B}T={10}^{-3}$.

Assume that we know, with some error, the size and the refractive index of the object that creates the phase difference. We can make an initial estimation of the phase difference between the two paths because it is proportional to the length travelled inside the object. We can model this situation assuming that $\theta$ is a random variable. As an example, we consider a Gaussian distribution centered at $\pi$, with standard deviation *π*/4 and trimmed at the edges (0 and 2*π*). Using RESM, we calculate the quantum Van Trees information for different values of $|\alpha |$, see Fig. 2(a). The line is obtained using Eq. (24) with $\rho (\xi )=|\phi (\xi )\rangle \langle \phi (\xi )|$ as an ansatz. The figure suggests that the family of POVMS proposed is a good ansatz. When $|\alpha |$ decreases, the Van Trees information decreases, but when $|\alpha |=0$ still ${\tilde{Z}}_{Q} > 0$, since an estimation of the phase difference can be done with the information we already have about the parameter prior to any measurements. In order to do the calculation we approximate the coherent states with a truncated Hilbert space and limited the number of outcomes of the POVMs. We observe the results as a function of Hilbert space dimension and the number of outcomes of the POVMs. We find that a Hilbert space of dimension 7 and a POVM of 10 outcomes give us stable results.

##### Initial displaced thermal state

As a final example, we consider estimating a parameter, chosen from a given distribution, encoded in a non-pure state. Again, there are no analytical expressions for the quantum Fisher information for this case. We calculate ${\tilde{Z}}_{Q}$ in order to bound the error in estimating the parameter. We build upon the last example, considering a thermal state displaced by the same operator that would give the state (31). Let

32 $$\rho (\theta ,T)={e}^{i\theta \hat{n}}[\hat{D}(\alpha ){\rho }_{th}(T){\hat{D}}^{\dagger }(\alpha )]{e}^{-i\theta \hat{n}}$$

with

33 $${\rho }_{th}(T)=\sum _{n}\,\frac{{\langle n\rangle }^{n}(T)}{{(1+\langle n\rangle (T))}^{n+1}}|n\rangle \langle n|$$

and

34 $$\langle n\rangle (T)={\left[\exp \left(\frac{\hslash \nu }{{k}_{B}T}\right)-1\right]}^{-1}=|\alpha {|}^{2}$$

be the state in which the parameter (*θ*) is encoded. For the Fig. 2(b), we used again a Gaussian distribution with mean $\pi$ and standard deviation *π*/4.

In Fig. 2(b) we show the numerical calculations of ${\tilde{Z}}_{Q}$ using the algorithm RESM. We compare it with the the ansatz composed of the two outcome POVM (24) with $\rho (\xi )=|\phi (\xi )\rangle \langle \phi (\xi )|$, see Eq. (32). We see that the points calculated with the RESM algorithm which are a lower bound of ${\tilde{Z}}_{Q}$ beat the ansatz case for most points. We expect such behavior as the state in consideration (32) is a mixed state.

## Discussion

The random extreme sampling method (RESM) can be used to find efficiently the maximum of a cost function over all possible quantum measurements. Particularly it is useful for finding limits in the precision of parameter estimation, through the cost function known as the quantum Fisher information, when the state to be measured is a mixed state. It can also be used to find the optimal measurement strategy by a given convex cost function by finding the POVM that maximizes it, at a considerable lower computational cost.

## Methods

The code implementation can be obtained in the repository^26^. To reproduce the results presented in 2.3.1, set the flag -o to Qubit and vary the flag --EtaAngle from 0 to *π*. For the results in sections 2.3.2 and 2.3.2, set the flag -o to CohPlusTherGaussian and to DispTherGaussian respectively. We also set the temperature with -T 0.001, the number of times to sample the space with -s 150 (or 200 for the displaced thermal state), the dimension of the Hilbert space to describe the system with --HilbertDim 7 and the number of outcomes of the POVM with --Outcomedim 10. For the pure state, as in 2.3.2, set -o CohPlusTherGaussian --MixConstant 1. The squared norm of *α* is set with the option --MeanPhotonNumb, which can be varied to reproduce the plots. The whole data set can be obtained with the command make all.
